# A Multidrug Resistance Plasmid pIMP26, Carrying *bla*_IMP-26_, *fosA5*, *bla*_DHA-1_, and *qnrB*4 in *Enterobacter cloacae*

**DOI:** 10.1038/s41598-019-46777-6

**Published:** 2019-07-15

**Authors:** Su Wang, Kaixin Zhou, Shuzhen Xiao, Lianyan Xie, Feifei Gu, Xinxin Li, Yuxing Ni, Jingyong Sun, Lizhong Han

**Affiliations:** Department of Clinical Microbiology, Ruijin Hospital, Shanghai Jiao Tong University School of Medicine, Shanghai, China

**Keywords:** Antimicrobial resistance, Bacterial genomics

## Abstract

IMP-26 was a rare IMP variant with more carbapenem-hydrolyzing activities, which was increasingly reported now in China. This study characterized a transferable multidrug resistance plasmid harboring *bla*_IMP-26_ from one *Enterobacter cloacae* bloodstream isolate in Shanghai and investigated the genetic environment of resistance genes. The isolate was subjected to antimicrobial susceptibility testing and multilocus sequence typing using broth microdilution method, Etest and PCR. The plasmid was analyzed through conjugation experiments, S1-nuclease pulsed-field gel electrophoresis and hybridization. Whole genome sequencing and sequence analysis was conducted for further investigation of the plasmid. *E. cloacae* RJ702, belonging to ST528 and carrying *bla*_IMP-26_, *bla*_DHA-1_, *qnrB*4 and *fosA5*, was resistant to almost all β-lactams, but susceptible to quinolones and tigecycline. The transconjugant inherited the multidrug resistance. The resistance genes were located on a 329,420-bp IncHI2 conjugative plasmid pIMP26 (ST1 subtype), which contained *trhK/trhV*, *tra*, *parA* and *stbA* family operon. The *bla*_IMP-26_ was arranged following *intI1*. The *bla*_DHA-1_ and *qnrB4* cluster was the downstream of IS*CR1*, same as that in p505108-MDR. The *fosA5* cassette was mediated by IS*4*. This was the first report on complete nucleotide of a *bla*_IMP-26_-carrying plasmid in *E. cloacae* in China. Plasmid pIMP26 hosted high phylogenetic mosaicism, transferability and plasticity.

## Introduction

Notoriously, extended and overuse of antibiotics have potentiated globally rapid emergence and spread of carbapenem-resistant *Enterobacterales* (CRE), posing a serious threat to clinical therapy and infection control^1–3^. The major driving force for the diversification and dissemination of CRE has been confirmed as the horizontal transfer of plasmid-mediated carbapenem-hydrolyzing enzymes (i.e., carbapenemase) genes^4^, among which the most prevalent and of particular clinical importance were *bla*_KPC_, *bla*_VIM_, *bla*_IMP_, *bla*_NDM_, and *bla*_OXA-48_^5^.

IMP, one kind of metallo-β-lactamases (MBLs), can efficiently inactivate almost β-lactams except monobactam^5^. IMP-1 was the first transferable MBL detected from *Pseudomonas aeruginosa* in Japan in 1991^6^; subsequently, the continuously clinical detection of *bla*_IMP-1_ in different species isolates in Japan^7,8^, as well as the discovery of IMP-2 in Italy^9^ and IMP-5 in Portugal^10^, marked the beginning of the upcoming flourish of IMP MBLs^11^. IMP-26 was first reported as an IMP-4 variant in Singapore in 2010 from a clinical carbapenem-resistant *P. aeruginosa* isolate by Koh TH *et al*.^12^. However, since then, there have been only sporadic reports on the IMP-26-production in Gram-negative bacilli^13–15^, especially in *Enterobacterales*^15^. Notably, isolates expressing IMP-26 were found significantly more resistant to doripenem and meropenem than that expressing IMP-1^13^.

*Enterobacter cloacae* was one member of the normal intestinal microflora of humans and animals, which has also assumed clinical importance and emerged as a major human pathogen causing hospital-acquired bacteremia, nosocomial pneumonia, urinary tract infections and so on^16,17^. In the past decade, the emergence of IMP-producing *E. cloacae* has been extensively reported as a challenge to clinical therapy because of its rapid worldwide transmission^14,16,18^. And in China, the most common IMP variants found in *E. cloacae* were IMP-8 and IMP-4^11,19,20^. As for IMP-26-producing *E. cloacae*, it has been only reported in Chongqing, Shanghai and Beijing worldwide^19,21,22^.

Our pilot study firstly reported two IMP-26-producing *E. cloacae* bloodstream isolates in Shanghai^21^. Considering the higher carbapenem-hydrolyzing activities and emerging reports in China of IMP-26, we subsequently analyzed the transferability and full nucleotide sequence of the corresponding multi-drug-resistance plasmid pIMP26 in this study, which carried several important resistance determinants, such as *bla*_IMP-26_, *bla*_DHA-1_, *aacA4*, *qnrB4* and *fosA5*, conferring resistance to carbapenems, cephalosporins, aminoglycoside and fosfomycin, respectively.

## Methods

### Isolate and antimicrobial susceptibility testing

Isolate RJ702 was obtained from the blood of a female patient with uterine malignancy at Ruijin Hospital in April 2013. The carbapenem-resistant isolate was first isolated at day 28 after admission. The previous travel history of the patient was not documented.

The initial species identification of RJ702 was performed using MALDI-TOF MS (bioMérieux, Marcy-l’Étoile, France). The minimum inhibitory concentrations (MICs) of ceftriaxone, ceftazidime, cefotaxime, cefepime, aztreonam, ciprofloxacin, levofloxacin, amikacin, gentamicin, piperacillin/tazobactam, cefoperazone/sulbactam, trimethoprim/sulfamethoxazole and tigecycline were determined using the broth microdilution method according to guidelines of the Clinical and Laboratory Standards Institute (CLSI M07-A9)^23^, while that of meropenem, ertapenem and imipenem were determined using the Etest (bioMérieux, Marcy-l’Étoile, France). The susceptibility results were interpreted according to the guidelines of CLSI M100-S25^24^; while the breakpoint for tigecycline was according to that of European Committee on Antimicrobial Susceptibility Testing (EUCAST) V6.0^25^. *Escherichia coli* ATCC25922 was used as the quality control. PCR was performed to detect the “big five” carbapenemase genes (*bla*_KPC_, *bla*_NDM_, *bla*_IMP_, *bla*_VIM_ and *bla*_OXA-48_).

### Multilocus sequence typing (MLST)

A MLST scheme was used to assign *E. cloacae* to clonal lineages, including seven housekeeping genes (*dnaA*, *fusA*, *gyrB*, *leuS*, *pyrG*, *rplB*, and *rpoB*) as described by Miyoshi-Akiyama^26^. The combination of seven alleles can define the sequence types (STs) on the MLST website (http://pubmlst.org/ecloacae/).

### Plasmid conjugation, S1-nuclease pulsed-field gel electrophoresis (S1-PFGE), and southern hybridization

The transferability of the resistance genes was assessed in broth culture using *E. coli* J53 Azr (sodiumazide-resistant) as the recipient. The transconjugants were selected on MacConkey agar containing sodiumazide (100 mg/L) and meropenem (2 mg/L) or ceftazidime (1 mg/L). PCR was employed to confirm the existence of *bla*_IMP-26_. DNA plugs of the parental and transconjugant digested with S1-nuclease were prepared and separated by PFGE, and then transferred to positively charged nylon membrane (Roche Applied Science, Germany). The membrane was hybridized with digoxigenin-labeled *bla*_IMP-26_ specific probes.

### DNA sequencing and genomics analysis

Genomic DNA of *E. cloacae* RJ702 was isolated using ChargeSwitch® gDNA Mini Bacteria Kit (Life Technologies, Carlsbad, CA, USA) and sequenced by a combination of PacBio RSII (Pacific Biosciences, Menlo Park, CA, USA) and Illumina Hiseq X10 (Illumina, San Diego, CA, USA) sequencing platforms. The assembly was produced firstly using a hybrid *de novo* assembly solution modified by Koren, in which a *de-Bruijn* based assembly algorithm and a CLR reads correction algorithm were integrated in “PacBioToCA with Celera Assembler” pipeline^27,28^. The final assembly generated a circular genome sequence with no gap existed. The precise species identification was established based on average nucleotide identity (ANI) between RJ702 and other type strains of *E. cloacae subsp*. using Orthologous ANI Tool (OAT) recommended by Lee I *et al*.^29^. Annotation of the genomic sequence and alignment with other similar sequences were carried out using the BLAST Ring Image Generator (BRIG)^30^ and SnapGene program v4.3.2. Open reading frames (ORFs) were identified using Glimmer version 3.02 (http://cbcb.umd.edu/software/glimmer/). ORFs less than 300-bp were discarded. Insertion elements and resistance genes were identified using ISFinder (https://www-is.biotoul.fr) and ResFinder (https://cge.cbs.dtu.dk/services/ResFinder). PlasmidFinder (https://cge.cbs.dtu.dk/services/PlasmidFinder) and pMLST (https://cge.cbs.dtu.dk/services/pMLST/) were employed to detect and type the plasmids. BLAST (http://blast.ncbi.nlm.nih.gov/Blast.cgi) was used to identify related plasmids carrying *bla*_IMP_ to guide PCR-based gap closure and Sanger sequencing to assemble contigs into complete plasmids.

### Nucleotide sequence accession number

The completely annotated sequence of pIMP26 in *E. cloacae* RJ702 has been deposited in GenBank database (Accession Number: MH399264).

### Ethics approval and informed consent

The study was approved by Ruijin Hospital Ethics Committee (Shanghai Jiao Tong University School of Medicine), and the Review Board exempted the requirements for informed consent as this study only focused on bacteria.

## Results

### Precise species identification

RJ702 was initially identified as *E. cloacae* or E. *asburiae* by MALDI-TOF MS. Additionally, RJ702 showed 95.2874% ANI value with *E. cloacae* ECNIH2 (NZ_CP008823) and only 87.8944% with *E. asburiae* ATCC35953 (NZ_CP011863). The ANI values of RJ702 with *E. cloacae subsp. cloacae* ATCC13047 (NC_014121) and *E. cloacae subsp. dissolvens* SDM (NC_018079) were 89.0906% and 87.9528% respectively. Therefore, RJ702 was precisely identified as *E. cloacae subsp. cloacae*.

### Antimicrobial resistance and MLST

*E. cloacae subsp. cloacae* RJ702 belonging to ST528, exhibited resistance to cephalosporins, monobactam, carbapenems, β-lactam/β-lactamase inhibitor combinations (only cefoperazone/sulbactam), aminoglycosides, trimethoprim/sulfamethoxazole, and tigecycline. The transconjugant inherited resistance to these antibiotics (Table 1).

**Table 1:** Antibiotic susceptibilities of *E. cloacae* RJ702 and its transconjugant.

Antibiotics	Minimal Inhibitory Concentrations (μg/ml)			
	RJ702		RJ702-1	
Ceftriaxone	>64	R	>64	R
Ceftazidime	>32	R	>32	R
Cefotaxime	>64	R	>64	R
Cefepime	32	R	16	R
Aztreonam	> = 64	R	16	R
Meropenem^a^	8	R	12	R
Ertapenem^a^	8	R	6	R
Imipenem^a^	4	R	4	R
Piperacillin/tazobactam	4/4	S	2/4	S
Cefoperazone/sulbactam	64/32	R	64/32	R
Trimethoprim/sulfamethoxazole	> = 2/38	R	> = 2/38	R
Ciprofloxacin	0.25	S	0.5	S
Levofloxacin	0.5	S	0.5	S
Amikacin	> = 64	R	> = 64	R
Gentamicin	> = 16	R	> = 16	R
Tigecycline	0.5	S	< = 0.13	S

^a^Antimicrobial susceptibility of carbapenems was determined by Etest.

### Conjugation, S1-PFGE, and southern hybridization

**Figure 1:**
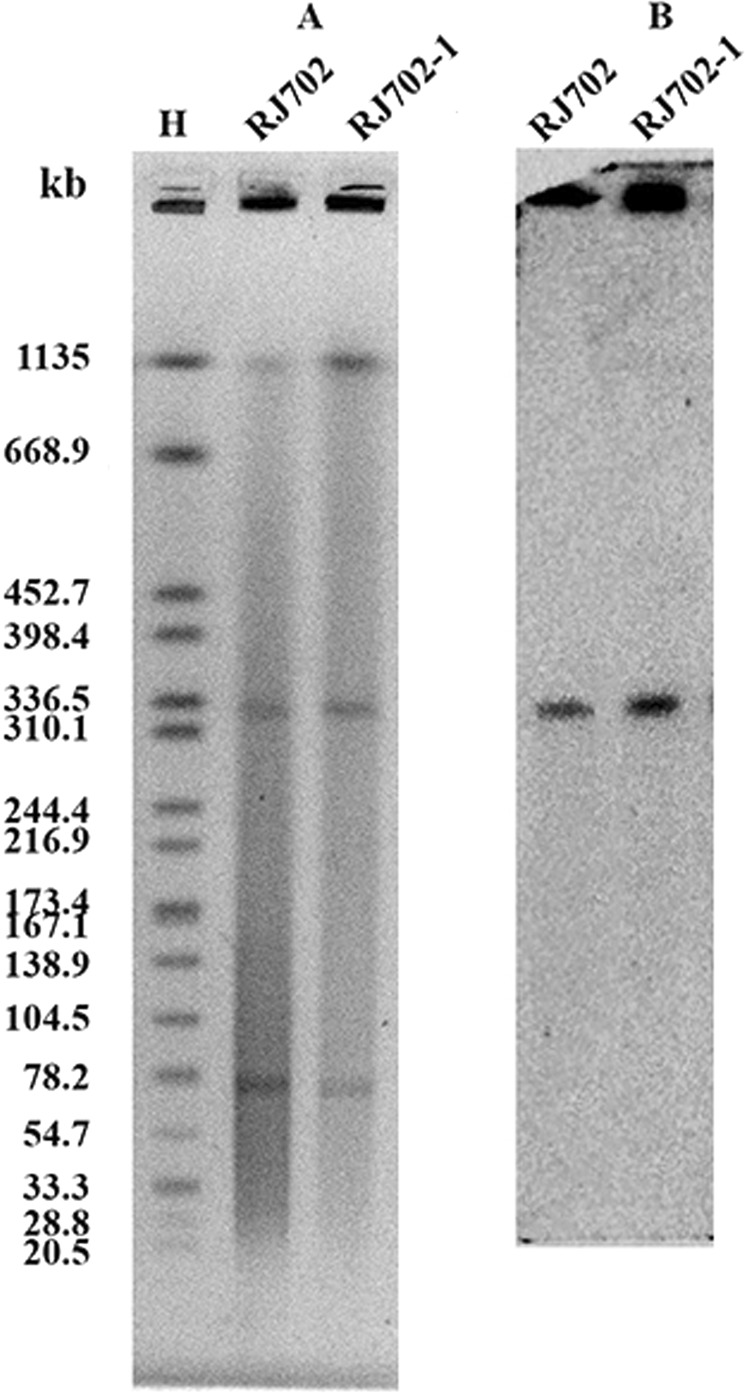
(**A**) The S1-PFGE profile of *E. cloacae* RJ702 and its transconjugant RJ702-1. M, *Salmonella enterica* serotype Braenderup H9812 was digested with *XbaI* as a molecular size marker. (**B**) The Southern blotting profile of *E. cloacae* RJ702 and its transconjugant RJ702-1 with *bla*_IMP-26_ specific probes.

The transconjugant RJ702-1 was obtained by plasmid conjugation experiments. S1-PFGE revealed that RJ702 harbored two plasmids (~320-kb and ~70-kb), and RJ702-1 inherited both. Southern hybridization analysis revealed *bla*_IMP-26_ located on the ~320-kb plasmid (pIMP26) (Fig. 1).

### Genome sequencing of RJ702

Whole genome sequencing generated 1,458,457 single reads and 4.70 Gb clean data total bases, which were *de novo* assembled to 184 contigs (75 > 1,000 bp; N50: 269,528 bp; N90: 45,440 bp). The bases in all contigs of RJ702 was 5.01 Mb with a 54.69% G + C content. The size of chromosome was 4,303,224 bp, and the bases in all contigs of two plasmids in RJ702 were 329,420 bp (pIMP26) and 78,322 bp respectively. PlasmidFinder presented that plasmid pIMP26 hosted two replicons, of which IncHI2 was 327 bp and IncHI2A was 630 bp; while the other plasmid hosted none.

### Backbones of pIMP26

**Figure 2:**
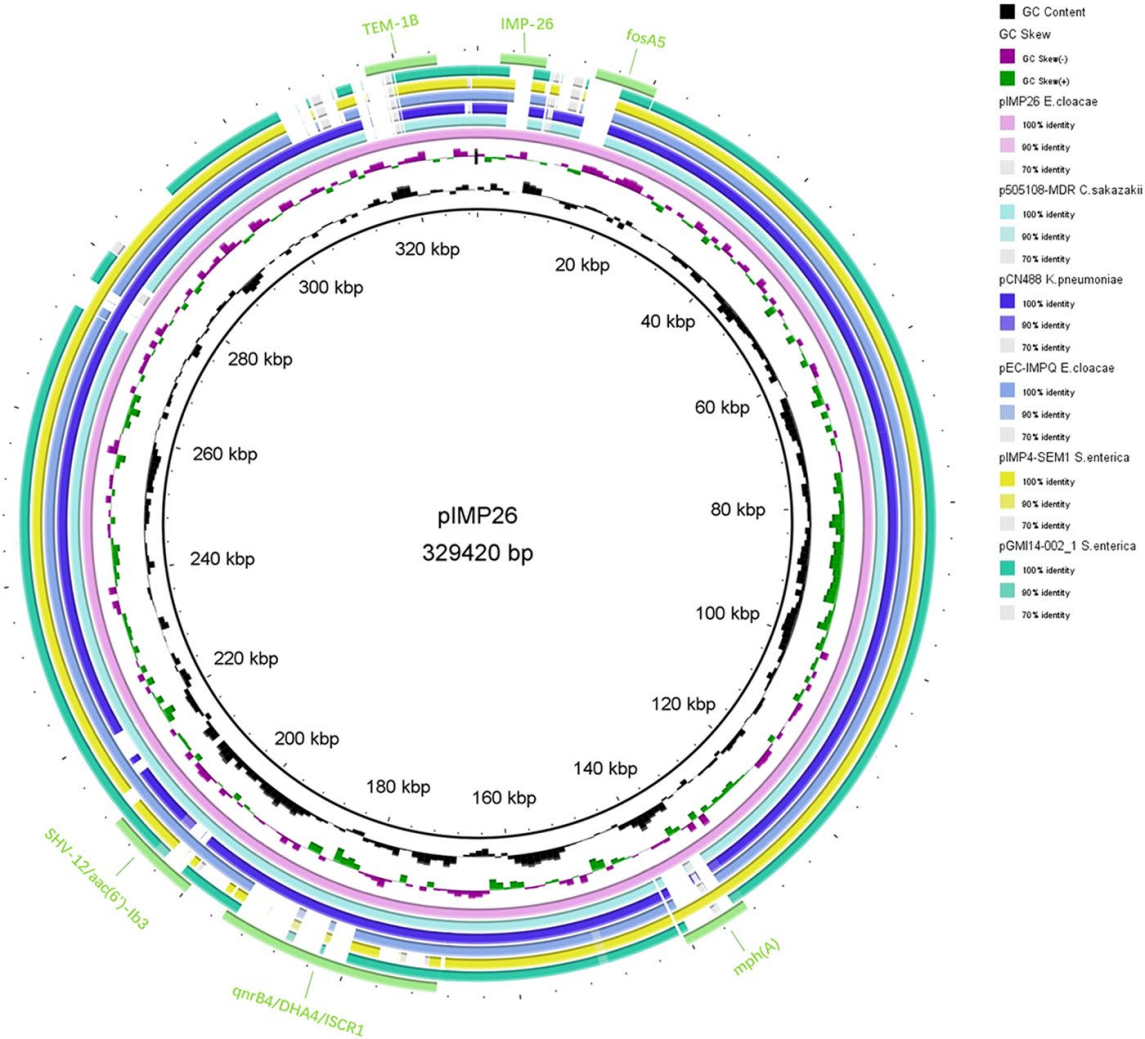
Circular map of plasmid pIMP26. The two inner circles represented the G + C content plotted against the average G + C content of 48.24% (black circle) and GC skew information (green and purple circles). Circles in different colors represented different plasmids (details in the legend), and the Genbank numbers were as follows: pIMP26 (MH399264), p505108-MDR (KY978628), pCNR48 (LT994835), pEC-IMPQ (EU855788), pIMP4-SEM1 (FJ384365), and pGMI14-002 (CP028197). The location of discussed resistance genes and *intI* were also demonstrated on the outer cyan-blue circle. The annotation of the genetic components were added manually using the Microsoft PowerPoint 2016 program.

Plasmid pIMP26 was a 329,420-bp closed circular DNA sequence with an average G + C content of 48.24% (Fig. 2). It hosted two *repB* replicons, and both belonged to IncHI2 prototype (ST1 subtype). BLAST searches indicated the backbone regions of pIMP26 highly similar to a 324,503-bp IncHI2 plasmid pEC-IMPQ (Genbank ID: EU855788) from an IMP-8-producing *E. cloacae* isolate in Taiwan (87% query coverage and 99% nucleotide identity) (Fig. 2). Annotation of the finished sequence data revealed that pIMP26 contained 381 ORFs, including *repB* (position: 32558–33613 and 47056–47931, for plasmid replication initiation), *trhK/trhV* (for stabilizing mating pairs during plasmid conjugation), four transfer gene clusters (locus *traB*, *traN*, *traG* and *traM*, encoding the conjugative apparatus), the *parA* family operon (for the replication/partitioning system with *repB*), and the *stbA* family operon (for plasmid stability).

### Resistance regions in pIMP26

**Figure 3:**
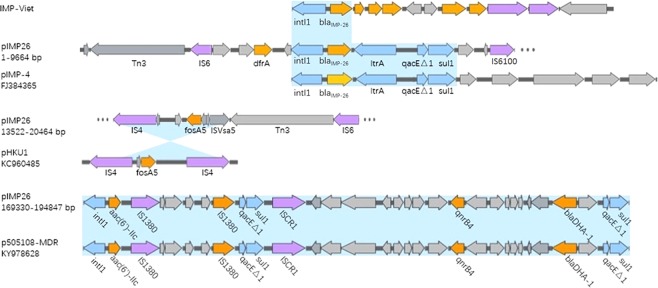
Plasmid accessory resistance regions. The comparison of linear DNA against the corresponding regions in different plasmids. The resistance genes were indicated by orange arrows and the insertion sequences are indicated by purple arrows. Shading regions denoted regions of homologous (>95% nucleotide identity).

Plasmid pIMP26 was rich in mobile genetic elements, including IS elements (IS*4*, IS*6*, IS*26*, IS*CR1*, etc.), transposons (Tn*3* family, etc.) and integrons (*intI1*, etc.), and contained multiple resistance genes (*bla*_IMP-26_, *fosA5*, *bla*_DHA-1_, *qnrB4*, *aac(*6′*)-Ib3*, *aac(6*′*)-IIc*, *aacA4*, *aph(6)-Id*, *strA*, *mph(A)*, *ere(A)*, *catA2*, *tet(D)*, *dfrA18*, *bla*_SHV-12_, two copies of *bla*_TEM-1B_, and three copies of *sul1*). According to BLAST searches (Fig. 3), the *bla*_IMP-26_ region was sequentially arranged as *intI1*, *bla*_IMP-26_, *ltrA*, *qacEΔ1* and *sul1* (position: 2567–7765), same as the *bla*_IMP-4_ cluster in pIMP-4 from an IMP-4-producing *Klebsiella pneumoniae* isolate in Shanghai (Genbank ID: FJ384365). In pIMP26, *bla*_IMP-26_ cassette is the downstream of IS*6* and followed by IS*6100* (IS*6*-like), *Eco128I* (type II restriction enzyme) and *M. EcoRII* (type II methyltransferase). The *fosA5* cluster was arranged sequentially as IS*4*, *RfaY*, *LysR*, *fosA5*, *RfaY*, IS*Vsa5* (IS*4*-like) (position: 13522–20464). The upper half of the cluster was opposite to that of pHKU1 from a *fosA5*-producing *E. coli* isolate in HK (Genbank ID: KC960485). The *bla*_DHA-1_ and *qnrB4* cluster was sequentially arranged as IS*CR1*, *sapC*, *sapB*, *sapA*, *YdeJ*, *qnrB4*, *pspE*, *pspA*, *pspB*, *pspC*, *pspD*, *LacI*, *bla*_DHA-1_, *LysR*, *hypA*, *qacEΔ1* and *sul1* (position: 169330–194847). It shared high similarity with p505108-MDR from a *bla*_DHA-1_- and *qnrB4*-producing *Cronobacter sakazakii* in China (Genbank ID: KY978628). Besides, two copies of *bla*_TEM-1B_ were both located in Tn*3* transposon in pIMP26.

## Discussion

The undesirable antibiotic resistance (especially carbapenem-resistance) has appeared and disseminated rapidly in Gram-negative bacilli, which was attributed largely to the acquisition of multiple resistance genes by horizontal plasmid-mediated genes transfer^4,5^. Our study was to map the genetic environment of a novel multi-drug-resistance plasmid pIMP26, in order to provide a new insight for the potential spread of *bla*_IMP-26_ and *fosA5* or correlations between genetic diagnosis and clinical treatment.

Firstly, the backbone of pIMP26 was blasted with different plasmids in BLAST. The origins of functional modules in pIMP26, such as multiple antibiotic resistance determinants, stably conjugal transfer (*tra* and *trh* family), mobile elements and plasmid maintenance (*stb* family) (Fig. 2), represented a strong transferability, stability and plasticity of this plasmid^31^. IncHI2 was one of the most prevalent broad-host-range plasmid families carrying different resistance determinants simultaneously in *Enterobacterales*^4,31,32^. As previously reported on *E. cloacae*, most β-lactamase-encoding genes (*bla*_SHV-12_, *bla*_CTX-M-15_, *bla*_NDM-1_, *bla*_IMP-4_, etc.) were also located on IncHI2 plasmids (subtype ST1) of 290~340-kb in size^18,31–35^, and our study also fit it. It should be noted that the similar backbone shared by pIMP26 and other plasmids (Fig. 2) in clinical isolates of *E. cloacae*, *K. pneumoniae* and *S. enterica* from different areas strongly suggested that inter-species genetic exchange also occurred, thus broadening the host range and dissemination of combined cargo genes. Besides, pIMP26 contained a wide variety of transposable elements carrying known antibiotic resistance genes. Tn*3* family transposon was the medium of TEM genes and *fosA5* was also located in Tn*3* in pIMP26 (Figs 2 and 3). The archetype of Tn*3* was known as some of the earliest unit transposons identified in Gram-negative bacilli. Tn*3* family members demonstrated transposition immunity, but homologous and/or *res*-mediated recombination between related elements can occur, creating hybrid elements^31^. And this would explain multiple Tn*3*-mediated resistance elements in pIMP26 in this study. However, further study is definitely needed to characterize the mechanisms behind the transfer or recombination of Tn*3*.

IMP-26, firstly found in *P. aeruginosa* in Singapore, was differed from IMP-4 at position 145 (G to T change); the translated amino acid sequence differed from IMP-4 at residue 49 (phenylalanine for valine)^12^. Blast searches indicated that the genetic structure surrounding *bla*_IMP-26_ has only revealed in a study from Vietnam up to now, containing *intI1*-*bla*_IMP-26_-*qacG*-*aac(6*′*)-Ib*-*orf3*-*orf4* (Fig. 2)^13^, and our study was the first time focusing on the complete nucleotide of the plasmid carrying *bla*_IMP-26_. Interesting was the *bla*_IMP-26_ region in pIMP26 different from that found in Vietnam (though both located on *intI1*)^13^; but same as the *bla*_IMP-4_ cluster of pIMP-4 in Shanghai (Genbank ID: FJ384365) (Fig. 3). It prompted that the *bla*_IMP-26_ detected in our study maybe originated from *bla*_IMP-4_ or the genetic mutation may occur during transfer of *bla*_IMP-26_ cassette.

The prevalence and dissemination of *fosA5* have probably been underestimated^36^. Previous study once found IS*10* playing an important role in the mobilization of *fosA5*^37^. However, the upper half consistent with pHKU1 in pIMP26 indicated that IS*4* might also related to its mobilization^36^ (Fig. 3). Plasmid carrying *bla*_DHA_ was usually reported also carrying *qnrB4*, *bla*_SHV-12_^38^. This suggested that the cassette in common of *qnrB4* and *bla*_DHA-1_ (Fig. 3) (including that in pIMP26) was derived from the same immediate ancestor. The *qnrB4*-*bla*_DHA_-containing region of pIMP26 was located after the 3′ conserved sequence (3′-CS) of *intI1* (Fig. 3), containing *aac(6*′*)-IIc*, *qacEΔ1* and *sul1*. Besides, an insertion sequence common region 1 (IS*CR1*) was identified downstream of *sul1*. IS*CR1* could mobilize the nearby sequence and a truncated 3′-CS from one integron to the 3′-CS of another integron through rolling-circle transposition, and provide a promoter for the expression of nearby genes^39^; this may lead to the co-carriage of multiple resistant genes in one plasmid and the multi-drug resistance of clinical isolates.

Interestingly, our study showed the *qnrB4*- and *aac(6*′*)-Ib3*-harboring RJ702 susceptible to quinolones (MIC = 0.5 or 0.25). We speculated that it was due to the absence of other mechanisms of chromosomal resistance (e.g. alterations in type II topoisomerases) in RJ702 other than plasmid-mediated quinolone resistant (PMQR) genes. Researchers found that PMQR mechanism caused only low-level quinolone-resistance on its own, which may not exceed the clinical breakpoints of susceptibility for quinolones but facilitated selections of higher-level resistance and posed threats to the treatment of infections by microorganisms hosting PMQR genes^40^, which could validate our speculation and underline the necessity of monitoring on PMQR genes.

This is the first report on the entire structure of *bla*_IMP-26_-carring plasmid. To some extent, our study evidenced the increasing clinical significance of IncHI2 replicons as resistance genes’ reservoirs and provided insights on the possibilities of further spread in China and highlighted the needs for intensive surveillance and precautions.

## Conclusions

We firstly reported here the complete nucleotide sequence of a plasmid carrying *bla*_IMP-26_, which was an IncHI2 replicon simultaneously encoding multidrug resistance determinants, including β-lactam (*bla*_IMP-26_, *bla*_DHA-1_, *bla*_SHV-12_, etc.), aminoglycoside (*aac(6*′*)-IIc*, *aacA4*, *aph(6)-Id*, etc.), fluoroquinolone (*qnrB4*, *aac(6*′*)-Ib3*) and fosfomycin (*fosA5*) resistance genes. New genetic context of *fosA5* was also characterized. The novel plasmid with multi-insertion of different resistant components and stable inheritance emphasized controlled use of clinical antibiotics to prevent selective pressure aggravating the emergence and dissemination of multi-drug resistance.

## Data Availability

The datasets used and/or analyzed during the current study are available from the corresponding author on reasonable request.
